# Thrombosis formation after COVID-19 vaccination Immunological Aspects: Review article

**DOI:** 10.1016/j.sjbs.2021.09.065

**Published:** 2021-09-30

**Authors:** Hisham Ali Waggiallah

**Affiliations:** Department of Medical Laboratory Sciences, College of Applied Medical Sciences, Prince Sattam Bin Abdulaziz University, Saudi Arabia

**Keywords:** Thrombosis, COVID-19, Vaccination, Immunological mechanism

## Abstract

COVID-19 deteriorates type II pneumocytes and damages the alveolar immunologic balancing process through the inadvertent activation of a sequence of localized and general inflammatory responses. Due to an aggregation of uncleaved angiotensin II, the stimulated inflammatory cells cause cytokines synthesis and secretion (cytokine storming). The cytokines cause the systemic inflammatory response syndrome (SIRS), leading to widespread tissue injuries. Consequently, pro-coagulant factors are activated which increases the microthrombi in different tissues, resulting in ischemia, multiple organ dysfunction syndrome, acute respiratory distress syndrome, and increased mortality.

Vaccines recipients (via virus vector technology) have reported the incidence of thrombocytopenia and peculiar thrombotic events. After vaccination, using sera from patients who experienced thrombocytopenia and thrombosis showed increased reactivity in anti-PF4/heparin enzyme immunoassays and substantial platelet-activating antibodies (positive). In some sera of individuals suffering from heparin-induced thrombocytopenia (HIT), it has been observed that platelet-activating antibodies resulting from vaccination tend to bind to non-complexed PF4 alone.

## 1 Introduction

The ongoing COVID-19 pandemic, resulting from SARS-CoV-2 or severe acute respiratory syndrome coronavirus 2, triggered nationwide shutdown in most nations around the world. Consequently, the world is facing a huge socioeconomic disaster and irreversible financial problem. In China, novel β CoV or SARS-CoV-2 was initially found in adults suffering from acute lower respiratory tract infection (LRTI) of uncertain origin (Chan et al., 2020). As no age group is immune to the new virus, patients aged 60 or above and those with co-morbidities showed extreme symptoms. Most people affected by the COVID-19 are either asymptomatic or have a mild type of virus strains. It is a highly infectious disease that is primarily transmitted through respiratory droplets and near contact with infected persons or polluted items. It also increases the risk of nosocomial infections (Cascella et al., 2020, WHO, 2020). The COVID-19 has a significantly low mortality rate but a much higher rate of infection compared to SARS disease (Ruan, 2020, Mahase et al., 2020).

While the COVID-19 infection is widely spread through respiratory droplets, it is possible to take a fecal-oral route. Apart from sputum and pharyngeal swabs, the virus was detected in feces (D’Amico et al., 2020). A positive COVID-19 nasopharyngeal swab had identified and verified the vertical transmission of SARS-CoV-2. The new virus has a median incubation time of 5.2 days and the majority of patients develop the disease within 11.5 to 15.5 days. Therefore, people who have been exposed to infection are advised to be in quarantine for 2 weeks (Li et al., 2020).

## 2 Covid-19 immunological mechanisms

SARS CoV-2 resembles SARS CoV; it has densely glycosylated spike (S) proteins S1 fraction with receptor-binding domain (RBD) attaching to the angiotensin-converting enzyme 2 receptor (ACE-2 R) with 10–20x greater affinity compared to SARS CoV (Wrapp et al., 2020). This receptor is primarily found in both types of human alveolar epithelial cells (type II > type I) (Zhou et al., 2020, Tian et al., 2020, Gui et al., 2017). It is expressed by endothelial cells along with gastrointestinal (esophageal and intestinal), epithelium, and cardiac myocytes (Meng et al., 2020, Xiong et al., 2020). After binding to ACE-2 R, the virus employs its unique polybasic S1/S2 protease cleavage site by inserting SPRR in the spike protein. Transmembrane protease serine (TMPRSS) displayed on host cells detects and cleaves the site for fusion protein (S2 fraction) exposure, enabling the viral membrane to fuse with that of the host cell (Meng et al., 2020). ACE-2 R, as well as TMPRSSs has also shown to be significantly co-expressed in absorptive enterocytes, upper esophageal epithelium, and alveolar type 2 pneumocytes. It implies that the new virus can enter the host through the alveolar epithelium along with the esophageal and intestinal epithelium. Therefore, the possible target tissue of the new virus could co-express ACE-2 R and TMPRSS (Meng et al., 2020). This membrane fusion allows viral RNA to be internalized into the host cell’s cytoplasm, where it replicates and translates to form new viral proteins. Viral aggregation is the last stage before liberating virions from infected cells. It comprises nucleocapsid (N) proteins that bind to RNA molecules. The viral aggregations are protected by membrane and envelop protein; consequently, virions with an ability to infect the surrounding cells are developed. SARS CoV-2 infection primarily impairs type II pneumocytes (play an important role in tissue repair and surfactant biosynthesis), causing increased surface tension and dyspnea. These attenuated type II pneumocytes inadvertently activate a cascade of local and systemic inflammatory responses that impairs the alveolar immunologic balance mechanism. Due to the accumulation of un-cleaved angiotensin II, the activated inflammatory cells lead to excessive cytokine synthesis and release (cytokine storm) (Barkauskas et al., 2013, Kroetz et al., 2015, Nabhan et al., 2018). This further causes systemic inflammatory response syndrome (SIRS) resulting in widespread tissue injury. Additionally, pro-coagulant factors are widely activated leading to an increase in microthrombi in various tissues/organs. Such phenomenon is demonstrated by the abundance of highly elevated pro-inflammatory cytokines in serious patients of the COVID-19. The cytokines include Interleukins 1-β, 1RA, 7, 8, 9, 10, b FGF2, GCSF, GMCSF, IFNγ, IP10, MCP1, MIP1α, MIP1β, PDGFβ, TNFα, and VEGFA) (Rothan and Byrareddy, 2020).

## 3 Types of vaccine

The COVID-19 vaccine will assist an individual in improving their immunity against the virus. Vaccines stimulate an immune response, helping the body learn how to fight against viruses in the future. Most vaccines use a whole virus for stimulating the immune system. Some may use virus fragments or genetic material containing instructions to produce specific proteins similar to the ones found in viruses. Similarly, various COVID-19 vaccines use S protein – a spike-like structure on the coronavirus surface. It aids the virus's entry into a person’s cells and initiates an infection. Various forms of the currently available COVID-19 vaccines are given below (Mayo Clinic Staff, 2021) (Table 1, Table 2).

**Table 1:** Types and Details of Some COVID-19 Vaccines.

**Vaccine**	**Immunological action**
1- Pfizer-BioNTech	The vaccine is composed of lipid particles that encourage the RNA delivery to host cells and allow the SARS-CoV-2 S antigen expression. It protects the body against the COVID-19 by promoting an immune response to the S antigen. (Dong et al., 2020, Anderson et al., 2020)
2- Moderna	The nucleoside-modified mRNA vaccine is produced in the form of lipid particles. It allows the nucleoside-modified mRNA and SARS-CoV-2 to be delivered and expressed, respectively. The vaccine stimulates an immune response to the S antigen, thus protecting against the COVID-19. The antibodies are unique to the SARS-CoV-2 virus that help defend against potential infection. (Dong et al., 2020, Anderson et al., 2020)
3- Johnson & Johnson’s Janssen	The vaccine developed by Johnson & Johnson employs viral vector technology. Adenovirus 26 is a common cold virus that has been genetically modified to infect cells but not replicate within them. It cannot spread throughout the body and can only transmit genetic instructions. Instead of being held in little lipid balls, the weakened virus injects the genetic instructions into arm cells. Here, they make the parts appear like a part of the coronavirus spike protein – the knob-shaped structure that the virus requires to bind to cells. It primarily encourages the immune system to fend off later infection in the body, thus confronting the coronavirus. (McCrone, 2021)
4-Vaxzevria (AstraZeneca)	Developed from another virus (adenovirus), it has been altered to include the gene responsible for producing the spike protein of the SARS-CoV-2. It is needed by the virus to penetrate the cells of the body. Once administered, this vaccine introduces the virus gene into the body cells. They would then use this gene for generating the spike protein. When the immune system recognizes it as a foreign body, it will develop antibodies and trigger T cells to attack it. (EMA, 2021a, EMA, 2021b)
5- Sputnik	A combined vector vaccine, Gam-COVID-Vac, is based on rAd type 5 (rAd5) and rAd type 26 (rAd26). These vectors contain the full-length glycoprotein S gene (rAd5-S and rAd26-S) of the SARS-CoV-2. It had been known that antigens transmitted by adenoviral vectors trigger cellular as well as humoral immunity following a single immunization. It can be employed as an emergency prophylaxis method during a pandemic. Moreover, the combination of two immunizations results in a immune response of longer timespan. (Logunov et al., 2021)
6- Sinopharm	It is an inactivated virus vaccine; the genetic material of the virus has been removed to prevent the onset of disease. As inactivated viruses cannot replicate within the body, higher doses of the vaccine are required. Adjuvants (molecules that activate the immune system) are often used to improve the immune response. Usually, inactivated virus vaccines only cause antibody-mediated immunity (not cell-mediated immunity). (WHO, 2021)

**Table 2:** Benefits and Drawbacks of Immunogens Used in Vaccines (WHO, 2021).

**Immunogen**	**Description**	**Benefits**	**Drawbacks**	**Examples**
Inactivated virus	Inactivated dead virus	Triggers powerful antibody response	Needs significant amount of virus and shows low or no cellular response	Influenza, hepatitis A, and rabies
Viral subunit	A pathogen-derived protein	Fewer side effects compared to the whole virus (swelling or redness at the injection site)	Complex process and may be poorly immunogenic	Influenza
Viral vector	Viral pathogen expressed on a safe virus, does not result in disease	Fast development, powerful cellular response, and easier to produce	Prior exposure to vector virus (eg. adenovirus) is likely to lower immunogenicity, certain vectors need boosting with another vector	Ebola
Nucleic acid	mRNA coding for a viral protein	Fast development and powerful cellular immunity	Reduced antibody response	COVID-19

## 4 Thrombosis associated with thrombocytopenia

Based on a national multicenter retrospective study conducted in China, at admission in COVID-19, the cases of thrombocytopenia (150 × 10^9^/L) were 36.2 % (Yang et al.,2020), close to SARS (40–45%) and MERS (36%). (Eastin et al., 2020) Thrombocytopenia has been generally considered as an indicator of the severity of disease, and a gradual decrease in platelet counts has been linked to a rise in mortality (Liao et al., 2020). Thrombocytopenia is commonly thought to be an indication of bleeding; however, the incidence of bleeding in COVID-19 is significantly lower than in Ebola and other hemorrhagic infections. According to Liao et al., only 3 out of 55 non-survivors had nonlethal hemorrhagic incidents (Liao et al., 2020). Bowles et al., 2020 also did not observe any clinically relevant hemorrhage in 35 patients suffering from the COVID-19 with a long-lasting activated partial-thromboplastin time (aPTT). This can be described by the coagulation disorder pattern in COVID-19 patients which is of a highly extreme hypercoagulable state instead of a hypocoagulable state (Bowles et al., 2020). The following pathways are considered to take part in SARS-CoV-2-induced thrombocytopenia: (1) a compromised hematopoietic microenvironment due to cytokine storm or systemic inflammation, including increased IL-6 – a general incidence in the COVID-19 infection (Novavax Inc, 2021) which may cause hematopoiesis inhibition (Leviet al., 2020). (2) Similar to several coronavirus infections causing thrombocytopenia, SARS-CoV-2 can directly cause infection in hematopoietic stem cells or megakaryocytes via angiotensin-converting enzyme 2 (ACE2), CD13, or CD66a (Valletta et al., 2020). (3) Antiviral antibodies that cross-react with hematopoietic cells and/or platelets including anti-adenovirus antibodies may interact with GPIIb/IIIa – an integrin on platelets (Amgalan and Othman, 2020). Chen et al. indicated impaired megakaryocyte maturation causes delayed-phase thrombocytopenia in patients suffering from the COVID-19 (Chen et al., 2020). (4) An autopsy of a non-survivor showed that disseminated intravascular coagulation and thrombotic microangiopathy led to elevated platelet usage (Bowles et al., 2020). (5) Splenic/hepatic macrophages are likely to scavenge the activated platelets. In reality, two different teams (Chen et al., 2019; Manne et al., 2020) independently showed platelets are hyper-activated in patients suffering from the COVID-19. The hyperreactivity of platelets can be explained partly by mitogen-activated protein kinase (MAPK) pathway activation (Chen et al., 2019; Manne et al., 2020). Like rhinovirus, influenza virus, and others, SARS-CoV-2 has direct interaction with platelets, modifying their function as well as quantity. SARS-CoV-2 as well as SARS-CoV have been shown to bind to ACE2. The former has at least ten times greater affinity for ACE2 than the latter (Zhang et al., 2020). A serine protease – transmembrane protease serine 2 (TMPRSS2) – facilitates the fusion of the SARS-CoV-2 cell membrane through the proteolytic cleavage and the spike protein activation. According to Zhang et al., high levels of TMPRSS2 and ACE2 are expressed by platelets. SARS-CoV-2 was also observed to directly trigger the activation and aggregation of platelets and encourage thrombosis (Manne et al., 2020).

## 5 Thrombosis mechanism in vaccination

### 5.1 AstraZeneca Vaccine

Vaccines are important to manage the SARS-CoV-2-induced COVID-19 pandemic. Based on randomized, blinded, and controlled trials, the European Medical Agency approved four vaccines: Comirnaty (Pfizer/BioNTech), a nucleoside modified mRNA COVID-19 vaccine; a recombinant adenovirus type 26 vector encoding SARS-CoV-2 spike glycoprotein COVID-19 Vaccine (Janssen); a recombinant adenoviral (ChAdOx1) vector encoding the spike protein antigen of SARS-CoV-2, AZD1222 (AstraZeneca); and an mRNA-based vaccine encapsulated in a lipid nanoparticle (Moderna). Nevertheless, after common vaccination with a recombinant adenoviral vector that encodes the spike protein antigen of the SARS-CoV-2 (AZD1222, AstraZeneca), there have been several records of certain vaccine recipients experiencing rare thrombotic events and thrombocytopenia (Wrapp et al., 2020).

The clinical image of patients with mild to significant complications of thrombocytopenia and thrombotic at irregular sites about a week following SARS-CoV-2 vaccination with AZD1222 indicates a clinically similar condition to a commonly known thrombotic disorder called heparin-induced thrombocytopenia (HIT). It results from platelet-activating antibodies recognizing the multimolecular complexes developed by cationic PF4 and anionic heparin (Greinacher et al., 2020).

Serological tests using sera from patients who experienced thrombocytopenia and thrombosis after vaccination revealed high reactivity in anti-PF4/heparin enzyme immunoassays and strong platelet-activating antibodies that are positive (Wrapp et al., 2020). These antibodies resulting from vaccination tend to bind only to non-complexed PF4, as observed in a few sera from patients with HIT (Greinacher, 2015). If they are anti-PF4 autoantibodies resulting from the strong inflammatory stimulation of vaccines, it indicates that the vaccine causes the platelet-activating antibodies. Patients are recommended to be treated with non-heparin anticoagulants including direct oral anticoagulants (rivaroxaban, apixaban) – approved without the need for prior heparin therapy. Moreover, they are commonly used to treat thrombosis. Furthermore, they have been prescribed for the HIT therapy (Wrapp et al., 2020).

In another study, the characteristic antibodies were found following the onset of anticoagulation therapy with low-molecular-weight heparin for thrombosis and thrombocytopenia that pose significant threat to life. With the finding of antibodies, clinicians were finding it difficult to decide which anticoagulant to use during this syndrome – typically correlated with heparin (Warkentin et al., 2020). However, after the introduction of concomitant treatment with intravenous immune globulin and prednisolone, there has been an increase in platelet counts. No clinical evidence indicated the growth of thrombosis (Warkentin et al., 2020). Furthermore, serious concerns arose that the administration of anticoagulation alternatives to low-molecular-weight heparin or heparin could aggravate the current intra-cerebral hemorrhage. Fondaparinux seems to have a relatively long half-life compared to low-molecular-weight heparin. Also, there is no well-established reversal strategy for factor Xa inhibitors. It is pertinent to note that the counts of platelets continuously increased in the patients in spite of the continued low-molecular-weight heparin therapy. It is possibly because of the effectiveness of timely treatment using intravenous immune globulin, demonstrated to be significantly effective in spontaneous HIT therapy (Schultz et al., 2021).

### 5.2 Johnson & Johnson’s Janssen

The European Medicine Agency (EMA) has examined eight cases of thrombosis associated with thrombocytopenia in individuals who got vaccinated Janssen's COVID-19 vaccine in the U.S. These extremely rare forms of thrombosis (with thrombocytopenia) involved venous thrombosis, mostly in odd locations including splanchnic vein thrombosis, cerebral venous sinus thrombosis, and arterial thrombosis along with a lethal consequence in one case. Within three weeks of vaccination, all cases were observed in individuals aged below 60, mostly in females. These cases were strikingly similar to those reported with AstraZeneca's COVID-19 vaccine, Vaxzevria. In terms of mechanism, the vaccine is considered to induce an immune response resulting in HIT-like disorder. In this stage, the pathophysiological mechanism is not well defined, and it is difficult to identify particular risk factors. Healthcare practitioners should know the clinical aspects of thromboembolism and thrombocytopenia to be able to provide treatments to patients at the earliest according to the available guidelines. Thrombosis associated with thrombocytopenia also necessitates specialized clinical management. For diagnosing and treating this disorder, healthcare practitioners should refer to relevant guidelines and/or consult specialists (e.g., hematologists and coagulation specialists) (Irani et al., 2019).

### 5.3 Pfizer-BioNTech

#### 5.3.1 Allergic reactions

In clinical trials, some of the adverse effects of the Pfizer-BioNTech COVID-19 Vaccine were discomfort, swelling, and redness at the injection site along with headache, weakness, chills, muscle pain, fever, joint pain, malaise, nausea, and lymphadenopathy.

#### 5.3.2 Adverse effects during the Post-Authorization period

After Pfizer-BioNTech COVID-19 Vaccine administration outside clinical trials, significant allergic reactions such as anaphylaxis along with other reactions of hypersensitivity (e.g., rash, urticaria, pruritus, and angioedema) have been reported, in addition to pain in the upper extremity, vomiting, and diarrhea. Additional side effects (sometimes severe) may emerge with the widespread vaccination with Pfizer-BioNTech COVID-19 Vaccine (EMA, 2021a, EMA, 2021b).

### 5.4 Moderna

The mRNA-1273 vaccine protocol and mechanism are safe; there were no unexpected trends of concern (FDA, 2021). Overall, mild local reactions to vaccination were observed. However, following the second dose, it showed moderate-to-severe systemic side effects including headache, arthralgia, myalgia, and nausea in nearly 50% of the mRNA-1273 respondents. These are considered to be adverse effects, starting from nearly 15 h post vaccination and resolving by day 2 in the majority of participants with no long-term consequences, (FDA, 2021) after one dose of mRNA-1273, the reactogenicity level was less than that of recombinant adjuvanted zoster vaccine (approved recently). The level was comparable following the second mRNA-1273 dose (Baden et al., 2021, Cunningham et al., 2016).

A possibility of acute hypersensitivity is occasionally found with vaccines. In the COVE study, there was no such risk, though it is restricted to find rare events because of the size of the trial sample. The anecdotal records of an insignificant overabundance of Bell's palsy in this trial along with the BNT162b2 vaccine trial led to serious concerns. This possibility warrants further investigation. ^46^ Following the administration of the mRNA-1273 vaccine, there was not any sign of improved respiratory disease after infection in the short term, a concern arose from animal models employed to evaluate the constructs of certain SARS as well as the Middle East respiratory syndrome (MERS) vaccines (Lal et al., 2015). In the histopathological review, a Th2-biased immune response along with eosinophilic pulmonary infiltration distinguish elevated respiratory disease. In advanced clinical trials, the preclinical testing of several SARS-CoV-2 vaccines such as mRNA-1273 showed a Th1-biased vaccine response with no pulmonary infiltrates (Agrawal et al., 2016, Corbett et al., 2020, Turner, 2021). It is unclear if mRNA-1273 vaccination results in severe disease after long-term virus exposure.

### 5.5 Sputnik V

In the examination of Sputnik V, Russia's Covid-19 vaccine, there were not any cases of the formation of blood thrombosis post-inoculation which has been attributed to the purification technology of the vaccine. Gamaleya Research Institute of Epidemiology and Microbiology, Moscow produced the Sputnik V viral vector vaccine and had similar clinical aspects as AstraZeneca and Johnson & Johnson’s vaccines. However, according to Gamaleya Institute researchers, in contrast to other vaccines, Sputnik V is developed using a 4-stage purification technology. As a result, no cerebral venous sinus thrombosis (CVST) cases were observed in clinical trials. Like the vaccines developed by AstraZeneca and J&J, vector vaccines are inactive cold viruses that are harmless. They are relatively easy to manage as compared to mRNA vaccines that must be stored at extremely low temperatures. The purification technology used in Sputnik V assists in obtaining a highly purified product such as the analysis of the presence of free DNA that undergoes mandatory control. Inadequate purification may be the cause of thrombosis in some patients who received other vaccines. In addition, using very high doses of target DNA/RNA is likely to lead to an adverse interaction between the antibodies of a patient. It results in thrombocytes with vaccine elements and/or free DNA/RNA that can produce a complex with the PF4 factor (Turner, 2021, Xia et al., 2020).

### 5.6 Sinopharm

In China, a study analyzed an investigational inactivated whole-virus COVID-19 vaccine for its immunogenicity and protection in healthy adult volunteers. Under different injection protocols, the vaccine under study was well received in every dose group, and there were not any vaccine-related severe adverse effects (Zhu et al., 2020a, Zhu et al., 2020b). The most frequent side effect was mild and self-limiting injection site pain. When compared to the findings of other candidate vaccines, the incidence rate of side effects (15.0% for all participants) was observed to be lower in the present study (Zhu et al., 2020a, Zhu et al., 2020b, Sahin et al., 2020, Mulligan et al., 2020, Jackson et al., 2020, Folegatti et al., 2020). All studies indicated that the intensity of side effects ranged from mild to moderate and was self-limiting. However, the vaccine group (mostly greater than 60% and 100% in some studies) showed greater incidence rates compared to the control group. As a result, the inactivated vaccine used in the present study indicates a better safety profile than the vaccines delivered through other platforms. In this study, the neutralizing antibody response was observed for 14 days following the injections, and the results indicated that the inactivated vaccine could successfully induce an immune response. In both stages, the findings demonstrated that a relatively gap (21 and 28 days) between the 1st and 2nd injections generated greater antibody responses than the schedule of a relatively short interval (14-day group). Following the second injection, the antibody titers began to rise and continued even after the third injection, showing a boosting injection requirement. The ideal interval from 1st to 2nd injections and the timing of booster injections of the inactivated vaccine is not yet known. However, it requires an in-depth assessment of the trial results with extended follow-up and different intervention groups (Zhu et al., 2020a, Zhu et al., 2020b).

The screening protocol (including serological and nucleic acid testing) was performed to ascertain that participants were not contaminated with SARS-CoV-2 before enrollment. Therefore, the vaccine (not natural infections) was most likely to produce the observed humoral immune response. Moreover, in the study region, any new COVID-19 cases were not identified and participants did not report the symptoms of SARS-CoV-2 infection during the experiment (Zhu et al., 2020a, Zhu et al., 2020b).

## 6 Conclusion

It can be concluded that the most significant cause of thrombosis after vaccination is an immunological mechanism with marked thrombocytopenia – vaccine-induced thrombocytopenia (VIT). Vaccinations using the viral carrier technique that carries the spike part of the COVID-19 lead to the formation of different forms of thrombosis. However, purification technology should be applied to overpass the harmful consequences of the vaccines. The remaining vaccinations using other technologies have not been related to thrombosis; however, the side effects are limited to the local immune reactions where the injection was given.

## Declaration of Competing Interest

The authors declare that they have no known competing financial interests or personal relationships that could have appeared to influence the work reported in this paper.
